# The complete chloroplast genome sequence of *Plagiogyria euphlebia*, a fascinating fern with important taxonomic significance

**DOI:** 10.1080/23802359.2020.1716640

**Published:** 2020-01-24

**Authors:** Xiaolin Yang, Yuening Zhu, Min Mao, Yifei Feng, Hui Wang, Yongfeng Hong, Zhen Wang, Yingjuan Su, Ting Wang

**Affiliations:** a School of Life Sciences, Sun Yat-sen University, Guangzhou, China;; b Fairy Lake Botanical Garden, Shenzhen, China;; c Research Institute of Sun Yat-sen University, Shenzhen, China;; d College of Life Sciences, South China Agricultural University, Guangzhou, China

**Keywords:** *Plagiogyria euphlebia*, chloroplast genome, phylogenetic analysis

## Abstract

It is the first report on the complete chloroplast genome of *Plagiogyria euphlebia*, a fascinating fern with important taxonomic significance. Its genome size is 161,046 bp with 43.5% GC content, including a large single copy (LSC) region (90,975 bp), a small single copy (SSC) region (21,441 bp), and a pair of inverted repeats (IRa and IRb) regions (24,315 bp). The cp genome has 133 genes involving 89 protein-coding genes, 33 tRNA genes, and three pseudogenes. ML tree reveals that *P. euphlebia* is sister to Cyatheales, especially closely related to *Cibotium barometz.*

*Plagiogyria euphlebia* (Kunze) Mett. is a fascinating species in monogeneric fern family Plagiogyriaceae (Zhang and Nooteboom 2013). It has fertile and sterile fronds with stripes of different lengths. As a circum-Pacific species, *P. euphlebia* habitats in the forest at an altitude of 600–1500 m only restricted in China, Bhutan, India, Japan, Korea, Myanmar, Nepal, Philippines, and Vietnam (Zhang and Nooteboom 2013). Different majority of species in genus *Plagiogyria,* this fern is diploid with chromosome base number *x*=130 (Zhang and Nooteboom 2013). It is difficult to distinguish this fern from other species with intermediate forms and hybridization in *Plagiogyria.* Moreover, there is huge controversy in classification and systematic position for Plagiogyriaceae due to discordance in morphological and molecular evidences such as sporangial development and analysis based on four protein-coding plastid loci (Smith 1995; Zhang and Nooteboom 1998; Korall et al. 2006; Cao et al. 2011; Wang et al. 2018). Hence, sequencing the complete chloroplast genome of *P. euphlebia* will contribute to settle these issues and further promote phylogenetic investigation.

We collected *P. euphlebia* from Shenzhen Fairy Lake Botanical Garden (22°34′43.1″N, 114°9′55.98″E), voucher specimen of which was stored at the Herbarium of Sun Yat-sen University (SYS; voucher: SS Liu 20161023). Total genomic DNA was extracted from fresh leaves using Tiangen Plant Genomic DNA Kit (Tiangen Biotech Co., Beijing, China) and was further broken into 300 bp fragments by Covaris M220 (Covaris, USA). After ligation, purification, and amplification, paired-end genomic library was constructed and sequenced in Illumina Hiseq 2500 platform (IIIumina Inc., San Diego, CA, USA). Raw data (2.66 G) were filtered by Trimmatotic v0.32 (Bolger et al. 2014) and high-quality clean data (2.54 G) were assembled into complete chloroplast sequence using Velvet v1.2.07 (Zerbino and Birney 2008). DOGMA (Wyman et al. 2004) and tRNAscan-SE (Schattner et al. 2005) were used to predict gene and RNA annotation, which was further confirmed through BLAST searches and manual correction of intron/exon boundaries. In order to verify the phylogenetic position of *P. euphlebia*, nine ferns including *Marsilea crenata* as outgroup were used to construct maximum-likelihood (ML) bootstrap analysis using RAxML v.8.2.12 with GTRGAMMAI model and 1000 replicates (Stamatakis 2014) based on MAFFT alignment of complete chloroplast genome sequences (Katoh and Standley 2013).

The complete chloroplast genome of *P. euphlebia* (GeneBank accession: MN027504) is a circular DNA of 161,046 bp in length with 43.5% overall GC content. As a typical quadripartite structure, it contains a large single copy (LSC) region (90,975 bp), a small single copy (SSC) region (21,441 bp), which were separated by a pair of inverted repeats (IRa and IRb) regions (24,315 bp). The cp genome has 133 genes including 89 protein-coding genes, 33 tRNA genes, and three pseudogenes. All of the genes are found as single copy excluding 13 duplicated protein-coding genes. In addition, 14 genes (*ndhB*, *rps16*, *atpF*, *rpoC1*, *petB*, *petD*, *ndhA*, *rpl16*, *rpl2*, *trnG-UCC*, *trnV-UAC*, *trnA-UGC*, *trnI-GAU* and *trnL-CAA*) contain one intron, whereas three genes (*ycf3*, *clpP* and *rps12*) have two introns. GC content in LSC, SSC, and IR regions were 43%, 42%, and 44%, respectively. ML tree reveals that *P. euphlebia* is sister to Cyatheales, especially closely related to *Cibotium barometz* (Figure 1). The determination of its complete chloroplast genome sequences will provide significant molecular information to illuminate the Plagiogyriaceae phylogenetic analysis.

**Figure 1.**
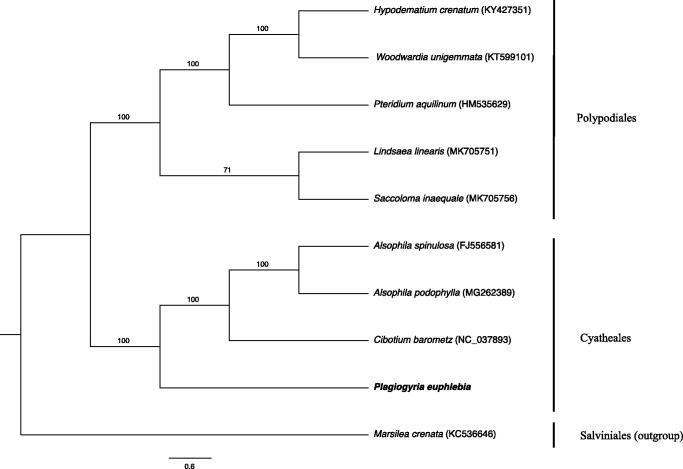
ML phylogenetic tree based on complete chloroplast genome of nine ferns including *Marsilea crenata* as outgroup. Node values indicate the bootstrap analysis with 1000 replicates.

## References

[CIT0001] Bolger AM, Lohse M, Usadel B. 2014. Trimmomatic: a flexible trimmer for Illumina sequence data. Bioinformatics. 30(15):2114–2120. 24695404 10.1093/bioinformatics/btu170 PMC4103590

[CIT0002] Cao JG, Dai XL, Wang QX. 2011. Archegonial development and oogenesis of the fern *Plagiogyria euphlebia* and their phylogenetic significance. Am Fern J. 101(4):231–240.

[CIT0003] Katoh K, Standley DM. 2013. MAFFT multiple sequence alignment software version 7: improvements in performance and usability. Mol Biol Evol. 30(4):772–780. 23329690 10.1093/molbev/mst010 PMC3603318

[CIT0004] Korall P, Pryer KM, Metzgar JS, Schneider H, Conant DS. 2006. Tree ferns: monophyletic groups and their relationships as revealed by four protein-coding plastid loci. Mol Phylogenet Evol. 39(3):830–845. 16481203 10.1016/j.ympev.2006.01.001

[CIT0005] Schattner P, Brooks AN, Lowe TM. 2005. The tRNAscan-SE, snoscan and snoGPS web servers for the detection of tRNAs and snoRNAs. Nucleic Acids Res. 33(suppl_2):W686–689. 15980563 10.1093/nar/gki366 PMC1160127

[CIT0006] Smith AR. 1995. Non-molecular phylogenetic hypotheses for ferns. Am Fern J. 85(4):104–122.

[CIT0007] Stamatakis A. 2014. RAxML version 8: a tool for phylogenetic analysis and post-analysis of large phylogenies. Bioinformatics. 30(9):1312–1313. 24451623 10.1093/bioinformatics/btu033 PMC3998144

[CIT0008] Wang RX, Shao W, Bai SM, Zhou ZP. 2018. Cytotaxonomic study of *Plagiogyria* (Plagiogyriaceae) from China. Flora. 243:53–57.

[CIT0009] Wyman SK, Jansen RK, Boore JL. 2004. Automatic annotation of organellar genomes with DOGMA. Bioinformatics. 20(17):3252–3255. 15180927 10.1093/bioinformatics/bth352

[CIT0010] Zerbino DR, Birney E. 2008. Velvet: algorithms for de novo short read assembly using de Bruijn graphs. Genome Res. 18(5):821–829. 18349386 10.1101/gr.074492.107 PMC2336801

[CIT0011] Zhang XC, Nooteboom PH. 1998. A taxonomic revision of Plagiogyriaceae. Blumea. 43:401–469.

[CIT0012] Zhang XC, Nooteboom PH. 2013. Plagiogyriaceae. In: Wu ZY, Raven PH, Hong DY, eds., Flora of China. Vol. 2–3 (Pteridophytes). Beijing: Science Press; p. 128–131.

